# Management and implications of severe COVID‐19 in pregnancy in the UK: data from the UK Obstetric Surveillance System national cohort

**DOI:** 10.1111/aogs.14329

**Published:** 2022-02-25

**Authors:** Nicola Vousden, Rema Ramakrishnan, Kathryn Bunch, Edward Morris, Nigel Simpson, Christopher Gale, Pat O’Brien, Maria Quigley, Peter Brocklehurst, Jennifer J. Kurinczuk, Marian Knight

**Affiliations:** ^1^ National Perinatal Epidemiology Unit, Nuffield Department of Population Health University of Oxford Oxford UK; ^2^ Royal College of Obstetricians and Gynaecologists London UK; ^3^ Department of Women’s and Children’s Health, School of Medicine University of Leeds Leeds UK; ^4^ Neonatal Medicine, School of Public Health, Faculty of Medicine Imperial College London London UK; ^5^ Institute for Women’s Health University College London London UK; ^6^ Birmingham Clinical Trials Unit, Institute of Applied Health Research University of Birmingham Birmingham UK

**Keywords:** adverse maternal and perinatal outcome, COVID‐19, population cohort, pregnancy

## Abstract

**Introduction:**

There is a lack of population level data on risk factors and impact of severe COVID‐19 in pregnancy. The aims of this study were to determine the characteristics, and maternal and perinatal outcomes associated with severe COVID‐19 in pregnancy compared with those with mild and moderate COVID‐19 and to explore the impact of timing of birth.

**Material and methods:**

This was a secondary analysis of a national, prospective cohort study. All pregnant women admitted to hospital in the UK with symptomatic SARS‐CoV‐2 from March 1, 2020 to October 31, 2021 were included. The severity of maternal infection (need for high flow or invasive ventilation, intensive care admission or died), pregnancy and perinatal outcomes, and the impact of timing of birth were analyzed using multivariable logistic regression.

**Results:**

Of 4436 pregnant women, 13.9% (*n* = 616) had severe infection. Women with severe infection were more likely to be aged ≥30 years (adjusted odds ratio [aOR] aged 30–39 1.48, 95% confidence interval [CI] 1.20–1.83), be overweight or obese (aOR 1.73, 95% CI 1.34–2.25 and aOR 2.52 95% CI 1.97–3.23, respectively), be of mixed ethnicity (aOR 1.93, 95% CI 1.17–3.21) or have gestational diabetes (aOR 1.43, 95% CI 1.09–1.87) compared with those with mild or moderate infection. Women with severe infection were more likely to have a pre‐labor cesarean birth (aOR 8.84, 95% CI 6.61–11.83), a very or extreme preterm birth (28–31+ weeks’ gestation, aOR 18.97, 95% CI 7.78–14.85; <28 weeks’ gestation, aOR 12.35, 95% CI 6.34–24.05) and their babies were more likely to be stillborn (aOR 2.51, 95% CI 1.35–4.66) or admitted to a neonatal unit (aOR 11.61, 95% CI 9.28–14.52). Of 112 women with severe infection who were discharged and gave birth at a later admission, the majority gave birth ≥36 weeks (85.7%), noting that three women in this group (2.7%) had a stillbirth.

**Conclusions:**

Severe COVID‐19 in pregnancy increases the risk of adverse outcomes. Information to promote uptake of vaccination should specifically target those at greatest risk of severe outcomes. Decisions about timing of birth should be informed by multidisciplinary team discussion; however, our data suggest that women with severe infection who do not require early delivery have mostly good outcomes but that those with severe infection at term may warrant rapid delivery.

**Abbreviations**
aOR: adjusted odds ratio
BMI: body mass index
CI: confidence interval
IQR: interquartile range
ITU: intensive care unit
NNU: neonatal unit
SARS‐CoV‐2: Severe Acute Respiratory Syndrome Coronavirus 2
UKOSS: UK Obstetric Surveillance System

**Key message**

Pregnant women who are ≥30 years old, overweight, of mixed ethnicity or have gestational diabetes have an increased risk of severe COVID‐19, which increases adverse outcomes for mother and baby. Information to promote vaccination should especially target these groups.

## 1 INTRODUCTION

Recent evidence suggests that approximately 8% of pregnant women with COVID‐19 on universal screening develop severe infection,^1^, ^2^, ^3^ with 3.2% admitted to an intensive care unit (ITU) and 9.2% having pneumonia.^1^ In addition to being at increased risk of any infection with SARS‐CoV‐2 in pregnancy, women aged >35 years (odds ratio [OR] 2.11), that are overweight (body mass index [BMI] >30 kg/m^2^; OR 2.71), are of non‐white ethnicity (OR 1.66) or that have any preexisting medical comorbidity (OR 1.70), have an increased risk of being admitted to ITU.^1^ These data were predominantly from single‐center cohorts and included a small number of women with severe outcomes. Evidence regarding the impact of gestational age on severity is also unclear. Although a systematic review reported from four studies that risk was not increased in the third trimester (*n* = 29),^1^ other studies have reported increased prevalence of symptomatic^4^ and severe infection in the later stages of pregnancy.^3^, ^5^

A high proportion (17%) of women with COVID‐19 in pregnancy are reported to have a preterm birth (<37 weeks of pregnancy), which reflects nearly a 50% increase compared with those without COVID‐19 (OR 1.47),^1^ and the odds of their baby being admitted to a neonatal unit (NNU) are also increased (OR 4.89).^1^ Evidence from subsequent small studies suggests that women with severe illness are more likely to have a preterm birth (41.8% vs 15.2%,^2^ 45.4% vs 5.2%^3^) and their babies more likely to be admitted to neonatal units (NNU, ie intensive or specialist care) than those with mild illness (50.4% vs 19.2%).^2^ However, neither study reported the degree of prematurity or relation to timing of infection. Current guidance states that the decision to initiate birth in order to facilitate maternal resuscitation, at the cost of greater prematurity, should be informed by individual assessment and multidisciplinary team discussion.^6^ However, there is a lack of robust data describing the clinical course of severe COVID‐19 in pregnancy and the impact of early delivery on maternal and neonatal morbidity and mortality.^7^

In the UK, the Delta variant of concern (B.1.617.2) became dominant in late May 2021^8^ and the proportion of women that experienced severe disease in pregnancy during this period was significantly increased (35.8% in Alpha‐dominant period vs 45.0% in Delta‐dominant period; adjusted odds ratio [aOR] 1.53).^9^ Therefore, improved knowledge on the risks and outcomes associated with severe infection is urgently required to inform clinical management and prevention policy. The aim of this study was to determine the incidence, characteristics, and maternal and perinatal outcomes of severe COVID‐19 in pregnant women admitted to hospital in the UK. The secondary aim was to explore the impact of timing of birth in relation to maternal and perinatal outcomes.

## 2 MATERIAL AND METHODS

This study was a secondary analysis of a prospective observational cohort study conducted using the UK Obstetric Surveillance System (UKOSS), across all 194 hospitals in the UK with a consultant‐led maternity unit.^10^ The methods have been extensively described.^9^ All pregnant women admitted to hospital with confirmed SARS‐CoV‐2 infection between March 1, 2020 and October 31, 2021, were notified to UKOSS. Reporters who had notified a case but not returned data, received email reminders at weekly intervals for 3 weeks. In addition to receipt of real‐time reports, zero reports were confirmed to ensure completeness.

Women were defined as having confirmed symptomatic SARS‐CoV‐2 if they were admitted to hospital during pregnancy or within 2 days of giving birth with symptoms of COVID‐19 (fever, cough, sore throat, breathlessness, headache, fatigue, limb or joint pain, vomiting, rhinorrhea, diarrhea, anosmia or SARS CoV‐2 pneumonia on imaging) and had a positive SARS‐CoV‐2 PCR test during their admission or in the 7 days prior to admission (Figure S1). Information on women who died or who had a stillbirth or neonatal death, was cross‐checked with MBRRACE‐UK, the organization that undertakes maternal and perinatal death surveillance in the UK.^11^

The primary outcome was a composite outcome indicating severity of SARS‐CoV‐2 infection as shown in Table 1. Categories were developed based on the World Health Organization criteria.^12^ Each of the components of these categories was also analyzed separately, as were pregnancy and perinatal outcomes including mode and gestation of birth, stillbirth, live birth, admission to NNU and neonatal death.

**TABLE 1:** Composite categories to describe severity of maternal infection

COVID‐19 severity	Criteria
Asymptomatic	No symptoms of SARS‐CoV‐2 at any time
Mild	Symptomatic *without* evidence of SARS‐CoV‐2 pneumonia on imaging *and* not requiring any respiratory support at any time
Moderate	Symptomatic *and* evidence of SARS CoV‐2 pneumonia on imaging *or* requiring non‐invasive respiratory support (≤15 L/min flow rate) *or* admission O_2_ saturations <95% (and not meeting criteria for severe)
Severe	Admission to ITU *and/or* treatment with high flow (>15 L/min), CPAP, invasive ventilation or ECMO *and/or* maternal death

Abbreviations: CPAP, continuous positive airway pressure; ECMO, extracorporeal membrane oxygenation.

### 2.1 Statistical analyses

Statistical analyses were performed using STATA version 15 (StataCorp). Unconditional logistic regression was used to identify characteristics associated with severe maternal and neonatal adverse outcome with COVID‐19. The hypothesized causal relations between SARS‐CoV‐2 variant and severity of infection were identified using a directed acyclic graph (DAG) informed by existing literature (Figure S2).^13^, ^14^ Based on this, the model was adjusted for sociodemographics (age, ethnicity, BMI and employment), presence of one or more medical comorbidities that may increase the risk of severe infection (Table S1), gestational diabetes, parity and variant dominant period (Wildtype SARS‐CoV‐2: March 1–November 30, 2020, Alpha variant: December 1, 2020–May 15, 2021, and Delta variant: May 16–October 31, 2021). There were insufficient data to include vaccine status as a covariate in multivariable models, as data were only collected from February 1, 2021. Neonatal outcomes were adjusted for the same covariates excluding parity. Age and BMI were included as ordered categorical variables in the model. Potential effect modifiers were identified as those with strong associations with maternal severity and few levels.^15^ Interaction terms were added and subsequent likelihood ratio testing on removal performed, with a *P* < 0.05 considered as evidence of significant interaction, with none found.

Exploratory analysis was undertaken to examine the impact of timing of birth on pregnancy and perinatal outcomes. Pregnancy and perinatal outcomes were compared for two groups of women: those whose birth was expedited due to maternal COVID‐19 disease and those who were discharged following their COVID‐19 admission while still pregnant and then gave birth at a later admission. Women who gave birth spontaneously at the time of their infection were excluded from this analysis. Additionally, logistic regression was used to compare the probability of neonatal adverse outcome (admission to NNU or neonatal death) between those with mild/moderate or severe COVID‐19, according to the number of days from diagnosis to delivery (divided into quartiles), by inclusion of gestation at time of COVID‐19 diagnosis (in weeks) and time to delivery as effect modifiers and adjustment for the final covariate model.

### 2.2 Ethical approval

This study was approved by the HRA NRES Committee East Midlands – Nottingham 1 on September 11, 2012 in anticipation of a pandemic (Ref. Number: 12/EM/0365). An amendment to activate the study for COVID‐19 was approved on March 2, 2020. Individual patient consent was not required for the collection of pseudo‐anonymized routine data.

## 3 RESULTS

Between March 1, 2020 and October 31, 2021, 4436 pregnant women were admitted to hospital across the UK with symptoms of confirmed SARS‐CoV‐2 infection. Overall, two‐thirds (65.4% *n* = 2903) of women had mild infection, 20.7% (*n* = 917) had moderate infection and 13.9% (*n* = 616) had severe infection.

The characteristics of women with severe compared with mild and moderate infection are shown in Table 2. After adjustment, women with severe infection were 48% more likely to be aged 30–39 years and 2.6‐fold more likely to be 40 years or older compared with those with mild or moderate infection. Overall, nearly two‐thirds of women were overweight or obese (65.8%, *n* = 2792). Women with severe infection were more than twice as likely to be obese compared with those with mild to moderate infection. Women with severe infection were also more likely to be of mixed ethnicity, with a nonsignificant increased odds of being from Black, Asian and other ethnic groups compared with being of white ethnicity (Table 2). Women with severe infection were 43% more likely to have gestational diabetes. Although there was not strong evidence of an association with one or more preexisting medical comorbidities after adjustment (aOR 1.24, 95% CI 0.97–1.59), women with severe infection had a more than 2‐fold increased odds of pre‐existing hypertension (aOR 2.20, 95% CI 1.24–3.89). Most women were admitted in the third trimester (76.9%, *n* = 2911 in mild or moderate group vs 69.3%, *n* = 419 in severe group), but women with severe infection were more likely to be admitted earlier in pregnancy than were women with mild or moderate infection (Table 2).

**TABLE 2:** Characteristics of pregnant women with confirmed severe COVID‐19 admitted to hospital in the UK compared with non‐severe symptomatic COVID‐19

Characteristic	Mild/moderate COVID‐19	Severe COVID‐19	OR (95% CI)	aOR^a^ (95% CI)
	*Total n* = 3820	*Total n* = 616		
Age (years)	*n* (%)	*n* (%)		
<20	68 (1.8)	6 (1.0)	0.76 (0.32–1.78)	0.79 (0.33–1.91)
20–29	1490 (39.2)	173 (28.1)	REF	REF
30–39	2019 (53.1)	362 (58.9)	1.54 (1.27–1.87)	1.48 (1.20–1.83)
≥40	228 (6.0)	74 (12.0)	2.80 (2.06–3.80)	2.57 (1.83–3.62)
Missing	15	1		
Body mass index (BMI)				
Underweight: <18.5 kg/m^2^	54 (1.5)	4 (0.7)	0.86 (0.31–2.42)	0.87 (0.30–2.50)
Normal: 18.5–24 kg/m^2^	1280 (35.0)	110 (19.0)	REF	REF
Overweight: 25–29 kg/m^2^	1147 (31.3)	181 (31.2)	1.84 (1.43–2.36)	1.73 (1.34–2.25)
Obese: 30 kg/m^2^ or greater	1179 (32.2)	285 (49.1)	2.81 (2.23–3.55)	2.52 (1.97–3.23)
Missing	160	36		
Ethnic group				
White	2219 (59.5)	323 (54.0)	REF	REF
Asian	873 (3.4)	144 (24.1)	1.13 (0.92–1.40)	1.15 (0.91–1.46)
Black	389 (10.4)	75 (12.5)	1.32 (1.01–1.74)	1.22 (0.91–1.63)
Other	163 (4.4)	33 (5.5)	1.39 (0.94–2.06)	1.42 (0.92–2.19)
Mixed	83 (2.2)	23 (3.9)	1.90 (1.18–3.07)	1.93 (1.17–3.21)
Missing	93	18		
Current smoking	359 (9.7)	43 (7.3)	0.73 (0.53–1.02)	0.78 (0.55–1.11)
Missing	130	29		
One or more relevant preexisting medical problems	472 (12.4)	108 (17.5)	1.51 (1.20–1.90)	1.24 (0.97–1.59)
Asthma or chronic respiratory disease	330 (8.6)	73 (11.9)	1.42 (1.09–1.86)	0.95 (0.58–1.56)
Hypertension	59 (1.5)	27 (4.4)	2.92 (1.84–4.65)	2.20 (1.24–3.89)
Diabetes	67 (1.8)	16 (2.6)	1.49 (0.86–2.59)	0.98 (0.51–1.88)
Cardiac disease	45 (1.2)	8 (1.3)	NC	NC
Renal disease	28 (0.7)	1 (0.2)	NC	NC
Immunodeficiency	31 (0.8)	3 (0.5)	NC	NC
Metabolic disease	7 (0.2)	1 (0.2)	NC	NC
Gestational diabetes	344 (9.0)	95 (15.4)	1.84 (1.44–2.35)	1.43 (1.09–1.87)
Either woman or partner in paid work	2903 (76.0)	430 (69.8)	0.73 (0.61–0.88)	0.82 (0.66–1.01)
Multiparous	2414 (63.2)	423 (68.7)	1.29 (1.07–1.55)	0.93 (0.75–1.15)
Missing	38	9		
Multiple pregnancy	82 (2.2)	24 (3.9)	1.85 (1.16–2.94)	1.38 (0.81–2.34)
Gestation at admission (weeks)^b^				
<22	406 (10.7)	49 (8.1)	1.68 (1.12–2.51)	1.55 (1.00–2.39)
22–27+6	469 (12.4)	114 (18.8)	3.39 (2.41–4.76)	3.16 (2.20–4.53)
28–31+6	463 (12.2)	130 (21.5)	3.91 (2.80–5.46)	3.46 (2.43–4.93)
32–33+6	316 (8.4)	81 (13.4)	3.57 (2.48–5.14)	3.32 (2.26–4.88)
34–35+6	598 (15.8)	134 (22.2)	3.12 (2.24–4.33)	3.01 (2.13–4.25)
36–37+6	289 (7.6)	25 (4.1)	1.20 (0.74–1.97)	1.10 (0.66–1.85)
38–39+6	780 (20.6)	56 (9.3)	REF	REF
≥40	465 (12.3)	16 (2.6)	0.48 (0.27–0.85)	0.54 (0.30–0.97)
Missing	34	11		

Abbreviations: NC, not calculated due to data sparsity; REF, reference group.

^a^ Adjusted for: age, BMI, ethnicity, employment status, one or more medical comorbidities, parity, trimester at infection and gestational diabetes.

^b^ Adjusted for: age, BMI, ethnicity, employment status, one or more medical comorbidities, parity and gestational diabetes.

Of those where the primary reason for admission was known (78.6%, *n* = 3485), half (49.2%, *n* = 1716) were admitted due to COVID‐19, 25.7% (*n* = 895) for labor and birth, and 25.1% (*n* = 874) for other reasons. A greater proportion of the severe group had low oxygen saturation (<95%) on admission (38.4% vs 26.0% in the moderate group) (Table 3). Of those admitted on or after July 1, 2020, when guidance on pharmacological therapy was available, only a minority of women received treatment with one or more standard pharmacological therapies for COVID‐19 (42.7%, *n* = 235 in severe group and 26.3%, *n* = 211 in moderate group received one or more of an antiviral, Tocilizumab, maternal steroids and monoclonal antibodies). Of 1761 women whose vaccination status was collected, 38 (2.2%) had received their first dose of SARS‐CoV‐2 vaccination prior to their positive SARS‐CoV‐2 virology test and 16 (<1%) women were fully vaccinated. In total, six women who had received any vaccination dose had severe disease (five had a single vaccine dose and one had two doses).

**TABLE 3:** Respiratory and medical support needs of pregnant women with mild, moderate or severe COVID‐19 in pregnancy

	Mild	Moderate	Severe
	Total *n* = 2903	Total *n* = 917	Total *n* = 616
Primary reason for hospital admission was COVID‐19	625 (29.6)	617 (77.7)	474 (82.0)
Oxygen saturation measured on admission (Yes)	1521 (52.5)	715 (77.5)	510 (82.8)
Median oxygen saturation (IQR)	98 (97–99)	96 (94–98)	96 (93–97)
Oxygen saturation <95%	^a^	186 (26.0)	196 (38.4)
Evidence of pneumonia on imaging	^a^	685 (74.2)	491 (79.7)
Respiratory support required	^a^	509 (61.5)	580 (96.5)
Noninvasive oxygen (nasal cannulae, mask or non‐rebreathe mask at <15 L)	^a^	479 (100)	101 (17.9)
CPAP or high flow (>15 L)	^a^	^a^	215 (38.1)
Invasive ventilation	^a^	^a^	213 (37.7)
ECMO	^a^	^a^	36 (6.4)
Level not known	^a^	30	15
Intensive care received	^a^	^a^	529 (85.9)
Medical management total^b^ ^,^ ^c^	59 (2.5)	211 (26.3)	235 (42.7)
Antivirals total^c^	8 (0.3)	21 (2.3)	70 (11.4)
Tocilizumab^c^	0 (0)	5 (0.5)	55 (8.9)
Steroids for maternal indication^c^	59 (2.0)	201 (21.8)	178 (28.9)
Regeneron monoclonal antibodies^c^	1 (<0.1)	3 (0.3)	2 (0.3)
Recruited to the RECOVERY trial	21 (0.7)	39 (4.2)	56 (9.1)
Steroids for fetal lung maturation	280 (10.8)	164 (22.3)	312 (57.7)

*Note*: All values in *n* (%).

Abbreviations: CPAP, continuous positive airway pressure; ECMO, extracorporeal membrane oxygenation.

^a^ Included in definition of groups.

^b^ Any of the listed medications given for medical management of SARS‐CoV‐2: Antivirals, Tocilizumab, maternal steroids, monoclonal antibodies.

^c^ Analysis restricted to women admitted on or after July 1, 2020, as guidance on medical management was published in June 2020.

Women with severe infection were more likely to give birth early, with nearly a quarter giving birth prior to 32 weeks of pregnancy (22.6%, *n* = 120) compared with 2.7% (*n* = 88) of those with mild or moderate infection (Table 4) and more than half giving birth at less than 36 weeks. Women with severe infection had a 50‐fold increased risk of having their birth expedited due to their COVID‐19 infection, were more likely to give birth by pre‐labor cesarean section, and less likely to have labor induced (Table 4). There were 22 maternal deaths in this cohort of women with symptomatic COVID‐19, although several remained critically unwell in intensive care at the time of writing.

**TABLE 4:** Pregnancy outcomes for women with severe vs non‐severe‐symptomatic COVID‐19

Pregnancy outcomes	Mild/moderate	Severe	OR (95% CI)	aOR (95% CI)^a^
	*n* (%)	*n* (%)		
	Total *n* = 3820	Total *n* = 616		
Ongoing pregnancy	493 (12.9)	75 (12.2)	NC	NC
Pregnancy loss	67 (2.0)	10 (1.9)	NC	NC
Birth	3260 (85.3)	531 (86.2)	NC	NC
Gestation at end of pregnancy (weeks)				
<22	54 (1.6)	9 (1.7)	2.32 (1.11–4.83)	4.08 (1.73–9.62)
22–27^+6^	32 (0.1)	26 (4.9)	11.32 (6.50–19.72)	12.35 (6.34–24.05)
28–31^+6^	63 (1.9)	94 (17.8)	20.79 (14.26–30.31)	18.97 (12.62–28.51)
32–33^+6^	78 (2.4)	66 (12.5)	11.79 (8.03–17.31)	13.55 (8.91–20.61)
34–35^+6^	181 (5.5)	91 (17.3)	7.00 (5.07–9.66)	6.70 (4.74–9.48)
36–37^+6^	545 (16.6)	92 (17.5)	2.35 (1.74–3.17)	2.17 (1.59–2.98)
38–39^+6^	1421 (43.1)	102 (19.4)	REF	REF
40 or more	920 (27.9)	47 (8.9)	0.71 (0.50–1.02)	0.87 (0.59–1.27)
Median (IQR)	39 (38–40)	36 (32–38)	NC	NC
Missing	33	14		
Birth expedited due to COVID‐19	116 (4.6)	326 (67.5)	42.82 (32.80–55.89)	51.28 (37.56–70.02)
Missing	752	48		
Mode of birth				
Pre‐labor cesarean	945 (29.4)	398 (76.1)	8.62 (6.61–11.24)	8.84 (6.61–11.83)
Cesarean after labor onset	471 (14.6)	39 (7.5)	1.69 (1.13–2.54)	1.76 (1.12–2.75)
Operative vaginal	349 (10.9)	15 (2.9)	0.88 (0.50–1.55)	1.30 (0.72–2.36)
Unassisted vaginal	1453 (45.2)	71 (13.6)	REF	REF
Missing	42	8		
Labor induced	1249 (38.3)	85 (16.0)	0.31 (0.24–0.39)	0.28 (0.22–0.36)
Pre‐eclampsia	62 (1.6)	16 (2.6)	1.69 (0.96–2.95)	1.43 (0.77–2.62)
Maternal death	^b^	22 (3.6)	NC	NC

Abbreviations: IQR, interquartile range; NC, not calculated; REF, reference group.

^a^ Adjusted for: age, BMI, ethnicity, employment status, one or more medical comorbidities, parity, trimester at infection and gestational diabetes.

^b^ Included in definition.

Although the overall proportion of babies that were stillborn was small, there was a higher proportion of stillbirths in women with severe infection than those with mild or moderate infection (3.3% vs 1.2%, aOR 2.51, 95% CI 1.35–4.66) (Table 5). Babies born to mothers with severe infection were 12‐fold more likely to be admitted to the NNU compared with those born to mothers with mild to moderate infection. There were 10 neonatal deaths (Table 5). Despite the earlier gestations at diagnosis, women with severe infection had a median time from diagnosis to giving birth of 6 days (interquartile range [IQR] 3–14 days), shorter than in women with mild or moderate infection (12 days from diagnosis to birth, IQR: 2–53 days).

**TABLE 5:** Perinatal outcomes for women with severe vs non‐severe SARS‐CoV2 infection

Perinatal outcomes	Mild/moderate	Severe	OR (95% CI)	aOR (95% CI)^a^
	*n* (%)	*n* (%)		
	Total *n* = 3328	Total *n* = 550		
Stillbirth	41 (1.2)	18 (3.3)	2.71 (1.55–4.75)	2.51 (1.35–4.66)
Admission to neonatal unit	482 (14.7)	354 (66.7)	11.48 (9.36–14.08)	11.61 (9.28–14.52)
Neonatal death	7 (0.2)	3 (0.6)	NC	NC

Abbreviations: NC, not calculated.

^a^ Adjusted for: age, BMI, ethnicity, employment status, one or more medical comorbidities, trimester at infection and gestational diabetes.

There were 112 women with severe infection in the second or third trimester who were discharged while still pregnant and then gave birth during a later admission (Table 6); the majority of these women required respiratory support (98.4%, *n* = 61 in the second trimester and 87.8%, *n* = 43 in the third trimester infection group) and intensive care (90.3% *n* = 56 in the second trimester and 74.0%, *n* = 37 in the third trimester infection group). All gave birth a substantial number of days after diagnosis (median 99 [IQR 89–103] in the second trimester and 48 [IQR 22–64] in the third trimester infection group). The majority gave birth at or after 36 weeks’ (82.2%, *n* = 51 in the second trimester and 90.0%%, *n* = 45 in the third trimester infection group). A lower proportion were admitted to the NNU compared with the overall group with severe infection (23.8%, *n* = 15 in the second trimester and 25.0%, *n* = 12 in the third trimester infection group, vs 66.7%, *n* = 354 in the severe group overall). Note, however, that three babies of women discharged while still pregnant after severe third trimester infection were stillborn (2.7% of women discharged while still pregnant after severe infection).

**TABLE 6:** Pregnancy and perinatal outcomes in women with severe COVID‐19 in relation to timing of birth, stratified by trimester at time of infection^a^

	Birth expedited due to COVID‐19		Discharged pregnant after COVID‐19 admission and pregnancy outcome is known^b^	
	2nd trimester infection	3rd trimester infection	2nd trimester	3rd trimester
	*n* (%)	*n* (%)	*n* (%)	*n* (%)
	Total *n* = 35	Total *n* = 286	Total *n* = 62	Total *n* = 50
Oxygen saturation <95% on admission	16 (57.1)	93 (36.6)	26 (47.3)	14 (32.6)
Evidence of pneumonia on imaging	33 (94.3)	237 (82.9)	52 (83.9)	36 (72.0)
Respiratory support required	35 (100.0)	285 (100.0)	61 (98.4)	43 (87.8)
Non‐invasive oxygen (nasal canulae, mask or non‐rebreathe mask at ≤15 L)	2 (5.7)	39 (14.0)	12 (20.0)	8 (19.1)
CPAP or high flow (>15 L)	8 (22.8)	90 (32.3)	35 (58.3)	27 (64.3)
Invasive ventilation	24 (68.6)	128 (45.9)	10 (16.7)	6 (14.3)
ECMO	1 (2.9)	22 (7.9)	3 (5.0)	1 (2.4)
Level not known	0	6	1	1
Intensive care received	35 (100.0)	234 (81.8)	56 (90.3)	37 (74.0)
Days from diagnosis of COVID‐19 to birth (median, IQR)	9 (4–13)	5 (2–8)	99 (89–130)	48 (22–64)
Gestation at end of pregnancy (weeks)				
<22	1 (2.9)	0 (0)	0 (0)	0 (0)
22–27^+6^	17 (48.6)	0 (0)	0 (0)	0 (0)
28–31^+6^	14 (40.0)	66 (23.2)	4 (6.5)	1 (2.0)
32–33^+6^	3 (8.6)	52 (18.3)	2 (3.2)	1 (2.0)
34–35^+6^	0 (0)	70 (24.6)	5 (8.1)	3 (6.0
36–37^+6^	0 (0)	50 (17.6)	14 (22.6)	10 (20.0)
38–39^+6^	0 (0)	36 (12.7)	24 (38.7)	24 (48.0)
40 or more	0 (0)	10 (3.5)	13 (21.0)	11 (22.0)
Missing	0	2	0	0
Neonatal outcomes	*N* = 38	*N* = 298	*N* = 64	*N* = 51
Stillbirth	1 (2.6)	5 (1.7)	1 (1.6)	3 (5.9)
Admission to neonatal unit^c^	36 (97.3)	235 (80.2)	15 (23.8)	12 (25.0)

Abbreviations: CPAP, continuous positive airway pressure; ECMO, extracorporeal membrane oxygenation.

^a^ Seven missing expected date of delivery so trimester at infection not known.

^b^ 865 missing date of discharge, or not yet discharged so excluded from this analysis.

^c^ Proportion of live‐born babies.

**FIGURE 1:**
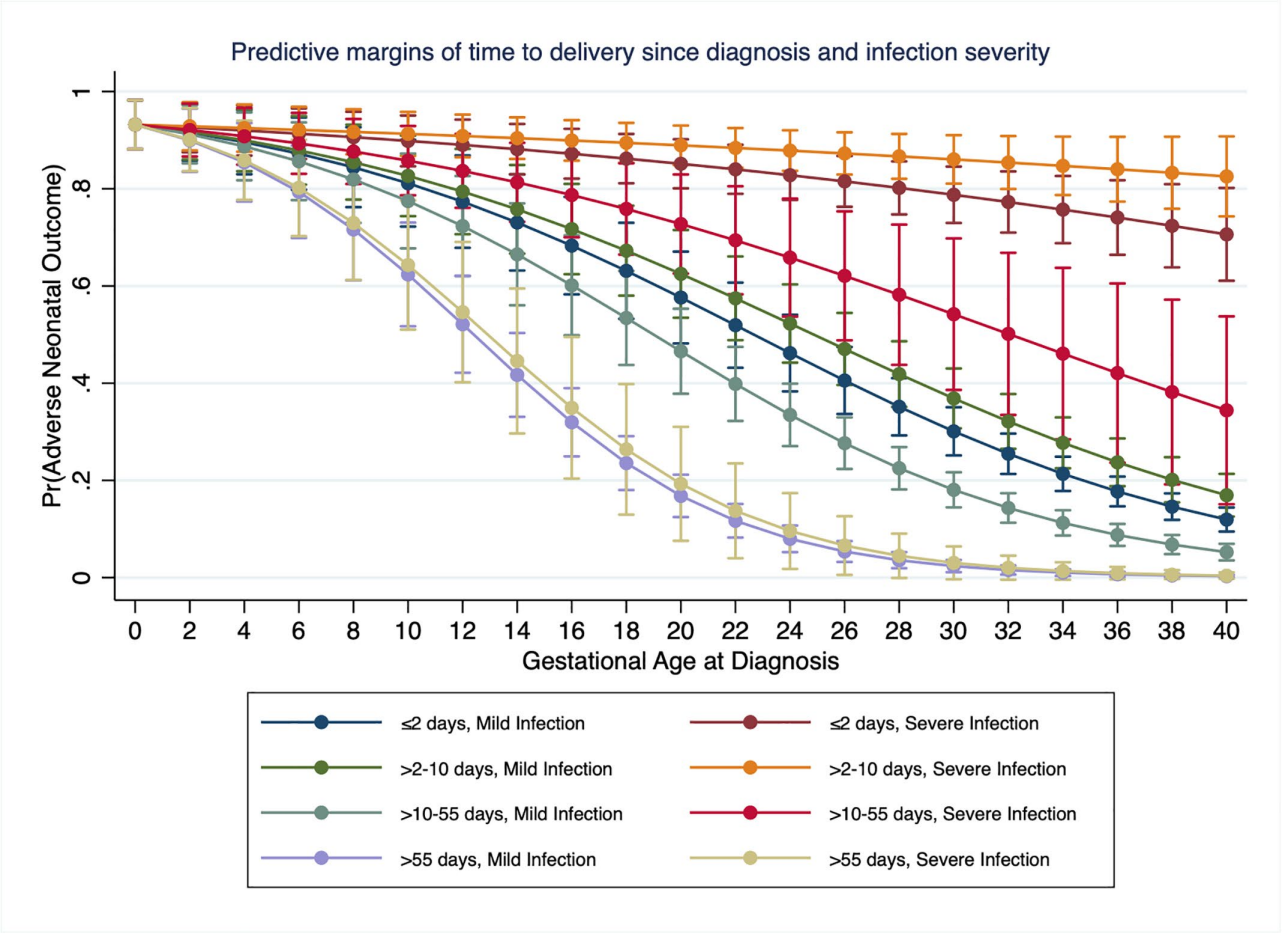
Predictive margins of time from diagnosis to birth and severity of infection across all gestational ages

The probabilities of neonatal adverse outcome (admission to NNU or neonatal death), taking into account the number of days from diagnosis of COVID‐19 to giving birth, the gestational age at diagnosis, and the severity of maternal infection are displayed in Figure 1. There is a correlation between the probability of neonatal adverse outcome and the gestational age at diagnosis, with reducing risk as gestational age increases, across all severity and timing of birth groups. Women that had more than 55 days from diagnosis to giving birth had a low probability of neonatal adverse outcome, regardless of severity of infection. In addition, there was a nonsignificant trend towards an increased probability of neonatal adverse outcome in women with severe infection who gave birth 3–10 days after diagnosis compared with those that give birth ≤2 days after diagnosis, suggesting that rapid delivery in women with severe infection at term may be beneficial.

## 4 DISCUSSION

This national cohort study demonstrated that between March 1, 2020 and October 31, 2021 there were 4436 pregnant women admitted to hospital across the UK with confirmed symptomatic COVID‐19, more than one in 10 of whom had severe infection. Women with severe infection were more likely to be aged ≥30 years, be overweight or obese, be of mixed ethnicity, have gestational diabetes and be admitted at earlier gestations than were women with mild or moderate COVID‐19.

A high proportion of women with severe infection had pneumonia, required respiratory support and were admitted to intensive care. However, the proportion of women that received treatment with standard pharmacological therapies for COVID‐19 was low. Women with severe infection were more likely to have extreme (<28 weeks’ gestation) and very (<32 weeks’ gestation) preterm births, with greatly increased risk of expedited birth due to SARS‐CoV‐2, more frequent pre‐labor cesarean section and less frequent induction of labor compared with women with mild or moderate infection. Women with severe infection had a 12‐fold increased risk of their babies being admitted to the NNU, in part due to the increase in expedited preterm birth. Most women with severe infection who were discharged still pregnant gave birth at term, with a lower proportion of their babies admitted to the NNU compared with those who gave birth at the time of their acute infection.

Key strengths of these data are the existing mechanism for national case identification of all women admitted to hospital and restriction to symptomatic cases only. This is because all obstetric admissions in the UK are screened for COVID‐19 and women screened are inherently more likely to experience an adverse outcome. For example, women attending hospital with preeclampsia will be screened for COVID‐19 and if incidentally found to be positive, their adverse outcome may incorrectly be attributed to COVID‐19. The large study size, with a large number of adverse outcomes, also improves reliability and generalizability compared with the existing literature. Although women with mild infection diagnosed and treated in the community will not be captured in this study, validation of cases of maternal and neonatal death against national surveillance systems reduces the risk of missing cases with severe adverse outcomes. Similarly, thresholds for hospital and ITU admission may vary by center and over time with changing knowledge of COVID‐19; however, it is highly likely that all those with severe COVID‐19 were included in this study. Detailed clinical information such as indication for expedited delivery and clinical condition after delivery was not collected in this national cohort and therefore could not be used to inform the thresholds of severe infection used in this analysis.

The characteristics associated with severe COVID‐19 in pregnancy are in keeping with those described in other studies.^1^, ^2^, ^3^, ^4^ We have shown a trend towards increased risk depending on ethnicity and employment status. Our data suggest that vaccination prevents admission to hospital with symptomatic COVID‐19 and against severe infection, but recent evidence suggests that uptake of vaccination in pregnancy is low and that women of black or Asian ethnicity compared with women of white ethnicity (aOR 0.37, 95% CI 0.10–1.06) and those living in deprived areas compared with those from more affluent areas (aOR 0.09, 95% CI 0.02–0.39) may be less likely to be vaccinated.^16^ This analysis therefore adds to the field by demonstrating the need to improve vaccine uptake and tackle misinformation in those at greatest risk of severe infection, especially where it is known that current vaccine uptake is low in these groups.

In keeping with other studies,^2^, ^3^ a high proportion of women with severe COVID‐19 have an iatrogenic preterm birth and evidence on its optimal timing in the presence of severe infection is therefore needed to inform practice. One further study, similar to this analysis, reported that median time to delivery in those with severe illness was less than in those with mild to moderate illness^2^; however, that analysis was limited by failure to report or take account of the gestation at infection. Although it is a strength that our analysis of timing of birth could take account of gestational age and severity of infection, this is likely still limited by residual confounding by indication, in that women with severe infection are most likely to be delivered rapidly regardless of gestation, with adverse impact on the baby, and it is therefore not possible to conclude the optimal timing of delivery in women with severe infection at preterm gestations. Although we provide new, largely reassuring data that the majority of outcomes for women who have severe infection and were discharged from hospital prior to giving birth were good, there was a small number of stillbirths in this group, emphasizing the importance of ongoing monitoring. In addition, taking account of time from diagnosis to delivery has highlighted a trend towards greater neonatal adverse outcomes in women with severe infection who gave birth 3–10 days after diagnosis compared with those that give birth ≤2 days after diagnosis. Therefore, we conclude that decisions around timing of birth are complex and need to be informed by multidisciplinary team discussion.

## 5 CONCLUSION

Severe COVID‐19 in pregnancy is relatively rare but it has a significant negative impact on both mother and baby. A very high proportion of women with severe COVID‐19 have a cesarean birth, with their babies born preterm and admitted to the NNU. Prevention of COVID‐19 is therefore key. This analysis identifies several characteristics that increase the risk of severe COVID‐19 in pregnancy and demonstrates promising vaccine efficacy. Current guidance in the UK recommends vaccination of all pregnant women, and this analysis demonstrates the need to specifically target misinformation and improve vaccine uptake in those identified as being at greatest risk. In keeping with the wider literature, most women admitted to hospital with COVID‐19 are in their third trimester; women should be informed of the greater risk at this time to allow consideration of earlier vaccination and measures to reduce exposure where possible. This analysis provides useful data that most women with severe infection who were discharged pregnant, went on to give birth at term with a low probability of neonatal adverse outcome, but that those with severe infection at term may warrant rapid delivery. Decisions about timing of birth should continue to be made following individual multidisciplinary team discussion.

## CONFLICT OF INTEREST

MK, MQ, PB, PO’B, JJK received grants from the NIHR in relation to the submitted work. EM is a Trustee of the Royal College of Obstetricians and Gynaecologists, British Menopause Society and Newly Chair of the Board of Trustees Group B Strep Support. CG was financially supported by the Medical Research Council through a Clinician Scientist Fellowship. PB was past chair of the MRC/NIHR Methodology Research Program panel with previous grant funding from MRC, NIHR and Welcome trust and provides consultancy services for personal fees to AG Biotest. KB, NV and NS have no conflicts of interest to declare.

## AUTHOR CONTRIBUTIONS

All authors contributed to the conceptualization, writing and editing of this study, had final approval of the version to be published and agree to be accountable for all aspects of the work. KB, EM, NS, CG, PO’B, MQ, PB, JJK and MK contributed to funding acquisition, supervision and methodology. NV, RR and MK contributed to data curation and formal analysis. All authors had access to verify the underlying data.

## Supporting information


Figures S1–S2
Table S1
