# Supplementary material for: Management and implications of severe COVID‐19 in pregnancy in the UK: data from the UK Obstetric Surveillance System national cohort
Source: Acta Obstet Gynecol Scand. 2022 Feb 25;101(4):461–70. doi: 10.1111/aogs.14329 (PMC9111211; doi:10.1111/aogs.14329)
Supplement: Supplementary file 1 — Figures S1–S2 Table S1 [file AOGS-101-461-s001.docx]

Supporting Figure S1: Flow chart of cases meeting case definition and included in the study from 01^st^ March 2020 to 31^st^ October 2021.

Supporting Figure S2: Directed acyclic graphs identifying covariates with potential causal pathways between SARS-CoV-2 infection in pregnancy and severe adverse outcome


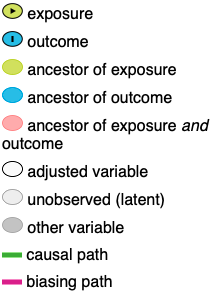

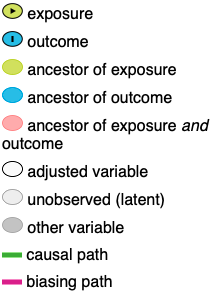

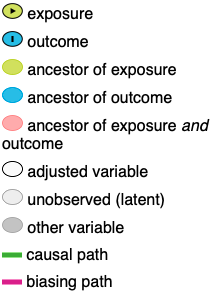


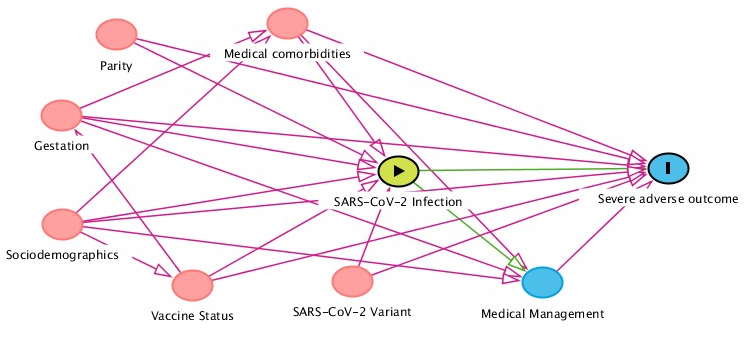


Supporting Table S1: Pre-existing medical comorbidities that may increase risk of COVID-19 and severity as informed by NHS guidance

| Relevant pre-existing comorbidities included in this study |
| --- |
| - Patients on or at risk of immunosuppression: - Solid organ transplant recipient on long term immune suppression treatment - Cancers with active treatments - Conditions that increase infection risk e.g., steroid treatment Systemic lupus erythematosus, Inflammatory bowel disease - Severe or chronic non severe respiratory disease e.g., Asthma - Rare diseases or inborn errors of metabolism e.g., Homozygous sickle cell disease - Significant or chronic cardiac disease - Chronic kidney disease - Diabetes mellitus: Type1 or Type2 |
